# The Medical School of London

**Published:** 1852-11

**Authors:** 


### THE MEDICAL SCHOOL OF LONDON.

Perhaps some notice of the various medical schools of the great metropolis of the world may be of interest to our readers. London, it is not to be disguised, has within a few years been absorbing much of the attention which so long centered about Paris, as at a still earlier period at Edinburgh. Of the great names of medicine, a large proportion is now to be found at London, and some details as to their local connections we think must prove attractive.

It is to be noticed, that the "EXAMINING BOARDS" in London are distinct from the schools. This desirable separation is one of the improvements so ardently urged by many advocates of medical reform among us, and a glance at the standard of "examinations," herewith subjoined, must certainly satisfy every candid inquirer, that a higher scale of requirements for a degree in medicine obtains under this system than our own.

The examining bodies in London are, the UNIVERSITY OF LONDON, the ROYAL COLLEGE OF PHYSICIANS, and the ROYAL COLLEGE OF SURGEONS. We give at length the list of Examiners and the regulations of these institutions.

First, the University of London, where in the Board of Examiners many celebrated names will be recognized :—

